# Weakly supervised pre-training for surgical step recognition using unannotated and heterogeneously labeled videos

**DOI:** 10.1007/s11548-025-03555-2

**Published:** 2025-12-02

**Authors:** Sreeram Kamabattula, Kai Chen, Kiran Bhattacharyya

**Affiliations:** Advanced Product Development, Intuitive Surgical, Inc., 5655 Spalding Drive, Peachtree Corners, GA 30092 USA

**Keywords:** Surgical step recognition, Weak supervision, Pre-training, Surgical video analysis, Minimally invasive surgery, Deep learning, Annotation efficiency

## Abstract

****Purpose**:**

Surgical video review is essential for minimally invasive surgical training, but manual annotation of surgical steps is time-consuming and limits scalability. We propose a weakly supervised pre-training framework that leverages unannotated or heterogeneously labeled surgical videos to improve automated surgical step recognition.

****Methods**:**

We evaluate three types of weak labels derived from unannotated datasets: (1) surgical phases from the same or other procedures, (2) surgical steps from different procedure types, and (3) intraoperative time progression. Using datasets from four robotic-assisted procedures (sleeve gastrectomy, hysterectomy, cholecystectomy, and radical prostatectomy), we simulate real-world annotation scarcity by varying the proportion of available step annotations ($\alpha$ $\in$ 0.25, 0.5, 0.75, 1.0). We benchmark the performance of a 2D CNN model trained with and without weak label pre-training.

****Results**:**

Pre-training with surgical phase labels—particularly from the same procedure type (Phase-Within)—consistently improved step recognition performance, with gains up to 6.4 f1-score points over standard ImageNet-based models under limited annotation conditions ($\alpha$ = 0.25 on SLG). Cross-procedure step pre-training was beneficial for some procedures, and time-based labels provided moderate gains depending on procedure structure. Label efficiency analysis shows the baseline model would require labeling an additional 30–60 videos at $\alpha$ = 0.25 to match the performance achieved by the best weak-pretraining strategy across procedures.

****Conclusion**:**

Weakly supervised pre-training offers a practical strategy to improve surgical step recognition when annotated data is scarce. This approach can support scalable feedback and assessment in surgical training workflows where comprehensive annotations are infeasible.

## Introduction

Video recordings from minimally invasive surgeries have become an essential resource for surgical education, performance evaluation, and skill acquisition [1]. Reviewing these videos with contextual annotations—such as surgical phases or steps—can enhance learning by helping trainees and educators identify critical moments and procedural structure [2, 3]. However, generating high-quality annotations is a time-consuming process that requires expert knowledge, which limits the scalability of automated video-based feedback systems [4].

Automated recognition of surgical activities using machine learning (ML) models offers a promising way to support scalable and context-aware surgical video analysis [5]. Yet, such models are fundamentally constrained by the volume and granularity of manually labeled training data. The challenge is further compounded by procedural variability: surgical activity annotations differ across procedure types, and may be labeled at different ontological levels (e.g., phases, steps, or gestures), each with varying levels of clinical specificity and annotation effort [6].

**Fig. 1:**
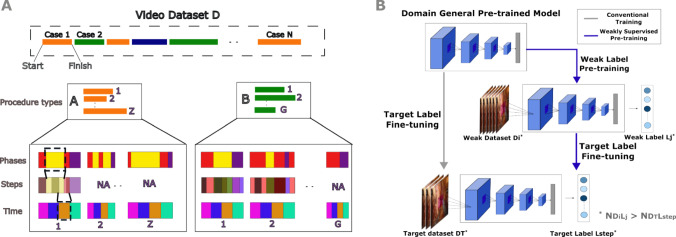
**A**) Surgical video dataset *D* contains cases from multiple procedure types (e.g., A and B), with each video potentially annotated at different ontological levels—surgical phases, steps, and automatically generated time bins. Not all videos have annotations for all label types, reflecting practical variability in real-world datasets. **B**) Our two-stage training framework begins with conventional pre-training on ImageNet to initialize a domain-general model. We then fine-tune this model using weak dataset-label pairs $\{(D_i, L_j)\}$, such as phases, elapsed time, or cross-procedural steps, selected to have greater label availability than the target dataset-label pair. The resulting model is further fine-tuned on limited step annotations for the target procedure to perform surgical step recognition

In particular, surgical steps—defined as procedure-specific actions with meaningful clinical goals in Meireles et al—are highly relevant for training and competency assessment [6–9]. However, their fine-grained nature makes them more difficult to annotate compared to higher-level labels such as phases, defined as the highest temporal level component of an operation in Meireles et al. [6, 9–11]. Consequently, large volumes of surgical video data remain unannotated or only partially labeled.

Prior work has explored weak supervision and self-supervised learning to reduce the annotation burden in surgical video analysis [12–18]. These approaches often focus on surgical phase recognition and rely on either synthetic labels or temporal coherence within unannotated videos [16, 19–21]. While effective, they are typically limited to single-procedure datasets or specific types of weak labels, and rarely examine surgical step recognition under real-world data sparsity conditions [22, 23].

**Table 1:** Surgical video datasets used for training and evaluation

Proc. Type	Abbrv.	# Videos	Steps Annotated?	Pre-train	Eval
Sleeve Gastrectomy	SLG	85	Yes	$\checkmark$	$\checkmark$
Hysterectomy	HYS	133	Yes	$\checkmark$	$\checkmark$
Cholecystectomy	CHO	329	Yes	$\checkmark$	$\checkmark$
Radical Prostatectomy	RPY	534	Yes	$\checkmark$ (step-RPY only)	N/A

Note that the Radical Prostatectomy (RPY) dataset was only used for pre-training with cross-procedure step labels (Step-RPY) since this is the largest dataset. Performance on step classification for RPY is not included in this study

**Table 2:** Summary of weak dataset-label pair scenarios for pre-training

Scenario	Label Type $\boldsymbol{L_j}$	Procedure Match?	Manual Annotation?	Description
*Phase-Within*	$L_{\text {phase}}$	Yes	Yes	Phases from same procedure
*Phase-All*	$L_{\text {phase}}$	Mixed	Yes	Phases from all procedures
*Step-RPY*	$L_{\text {step}}$	No (step-RPY only)	Yes	Steps from unrelated procedure
*Time-Within*	$L_{\text {time}}$	Yes	No	Elapsed time, same procedure
*Time-All*	$L_{\text {time}}$	Mixed	No	Elapsed time, all procedures

Note that Step-RPY pretraining only includes step labels from RPY

In this study, we systematically evaluate how different weak supervision strategies can enhance surgical step recognition. We propose a flexible pre-training and fine-tuning framework that leverages diverse sources of weak labels—including surgical phases, time progression, and steps from other procedure types for pre-training—to improve performance on surgical steps when annotated data for a target procedure is limited. Using datasets from four robotic-assisted procedures, we simulate varying annotation conditions and benchmark the impact of each pre-training strategy on downstream step recognition (Fig. 1).

Our findings show that certain weak label sources—particularly surgical phases from the same procedure—can significantly improve model performance even when only 25% videos in the dataset have surgical steps annotated. We discuss the relevance of each strategy, implications for clinical deployment, and future opportunities for scalable, low-cost annotation in surgical AI.

## Methods

### Problem formulation

We aim to improve automated recognition of procedure-specific *surgical steps* using video-based machine learning, particularly under conditions of limited annotated data. Given a surgical video dataset $D$, partitioned by procedure type and containing a mixture of annotated and unannotated videos, models for surgical step recognition typically rely solely on the annotated data from the target procedure type. However, this traditional approach does not take advantage of the remaining unannotated data within the target procedure, or any data from other procedure types, or other heterogenous labels that may be available such as phases. Recognizing this gap, we propose a weakly supervised pre-training approach that transfers knowledge from auxiliary datasets with *different labels or procedures* to improve step recognition.

Our objective is to maximize model performance on a target surgical step label $L_{\text {step}}$ for a given procedure data $D_T$, when only a fraction $\alpha$ of that dataset is annotated with steps. To do this, we first pre-train model on a weak dataset-label pair $(D_i, L_j)$ where either:

- $D_i \ne D_T$: different procedure type, or
- $L_j \ne L_{\text {step}}$: different label, or
- the data is unannotated (but temporally structured).

and later fine-tune on $(D_T, L_{\text {step}})$.

### Datasets and label types

We use robotic-assisted surgical videos from four procedures shown in Table 1. We consider three target procedures SLG, HYS, and CHO for evaluating surgical step recognition, while RPY is used exclusively for weak pre-training supervision.

Each dataset is annotated with surgical steps. From these annotations, we derive phase labels. Elapsed time labels are automatically annotated based on video timestamps.

- **Surgical steps** ($L_{\text {step}}$): Fine-grained, procedure-specific actions annotated by clinical experts. Each case is annotated by a single expert.
- **Surgical phases** ($L_{\text {phase}}$): Coarse-grained stages in the procedure that are easier to annotate. Since each surgical step maps to a unique surgical phase (many-to-one), we derive phase labels automatically from collected step annotations and a step-to-phase mapping [9]. Definitions of surgical phases do not allow for gaps between them, so any temporal gaps between successive step annotations 1) belonging to the same phase were annotated as belonging to that phase 2) belonging to different phases were back-filled by the phase of the first step.
- **Elapsed time** ($L_{\text {time}}$): Automatically derived by dividing the duration of each case into 10 equal temporal segments serving as a weak proxy for monotonic progression. Note that this is different from case progression which can be nonlinear and correspond to specific surgical events. However, equal time bins are a scalable proxy for progression which is equivalent across procedure types.

Specifically, we use the definitions of surgical phase and step as described in Meireles et al.[6]. For explicit examples of the annotation ontology, reports of inter-annotator variability, and the general framework used to develop the annotation ontology, please refer to Mlambo et al .[9].

### Weak supervision scenarios

We define five weakly supervised pre-training configurations shown in Table 2:

- Phase-Within: Pre-train on only target procedure type data using phase labels. This investigates the benefit of learning broader contextual information from the same procedure type for surgical step recognition.
- Phase-All: Pre-train using phase labels across all procedures (HYS + SLG + CHO) data, to assess whether broader context in general enhances model generalization.
- Time-Within: Leverage elapsed time labels within the target procedure for pre-training. This explores whether understanding temporal progression of the procedure can act as an effective weak supervisory signal for step recognition in scenarios where no other ontological labels (e.g. phases) are available.
- Time-All: Leverage elapsed time labels across all procedures data for pre-training.
- Cross-Procedure Step (Step-RPY): Pre-train on radical prostatectomy dataset using step labels, to evaluate the potential benefit of leveraging data from cross-procedures that contain extensive step annotations.

In these weak supervision scenarios, three pre-training configurations—Phase-All, Time-All, and Step-RPY—provide consistent weak pre-trained models for any target procedure (whether HYS, SLG, or CHO) step recognition. However, Phase-Within and Time-Within configurations are specific to individual procedure types, using only the same procedure’s data and labels for pre-training.

### Simulation of annotated data conditions

In practical clinical settings, only a small portion of surgical video data is often annotated with surgical steps. Thus, to simulate realistic annotation constraints, we define four levels of label availability for surgical steps in our datasets:

$$ \alpha \in \{0.25, 0.5, 0.75, 1.0\} $$

where $\alpha$ represents the proportion of the target dataset annotated with step labels.

**Table 3:** Number of step annotated videos for different $\alpha$

Proc. Type	0.25	0.5	0.75	1.0
Sleeve Gastrectomy	17	34	51	68
Hysterectomy	26	53	79	106
Cholecystectomy	66	132	197	263

For each target procedure (SLG, HYS, CHO), 20% of the videos are held out as a test set. From the remaining 80%, we subsample a fraction ($\alpha )$ of the videos to serve as the set labeled with steps and treat the rest as unannotated. This process is repeated over five random splits. The number of labeled videos under different $\alpha$ for each dataset is shown in Table 3. Note that we only simulate different levels of annotated data for surgical steps, and for all weak pre-training we consider phases, elapsed time, Step-RPY labels are available for the entire dataset.

### Model architecture and training procedure

We use the EfficientNet-V2S convolutional neural network [24], pre-trained on ImageNet [25], for both weak pre-training and target fine-tuning stages. Video frames are extracted at 1 fps and resized to $224 \times 224 \times 3$ following standard practices in [12, 26]. From the labeled set above, we consider 70% for training and 30% for validation. Training parameters include:

- Batch size: 64
- Learning rate: 0.001
- Loss function: Cross-entropy with weighted sampling to address class imbalance
- Early stopping: Based on validation loss
- Fine-tuning: All weights unfrozen for both stages

In total we train 9 individual weak pre-trained models, where 3 of them serve as common (Phase-all, Time-All, Step-RPY) pre-trained models for fine-tuning any target procedure, and two models (Phase-Within, Time-Within) are tailored to each target procedure. The corresponding weak pre-trained models are selected based on the target procedure type, and further fine-tuned for step recognition specific to each $\alpha$ labeled data.

**Fig. 2:**
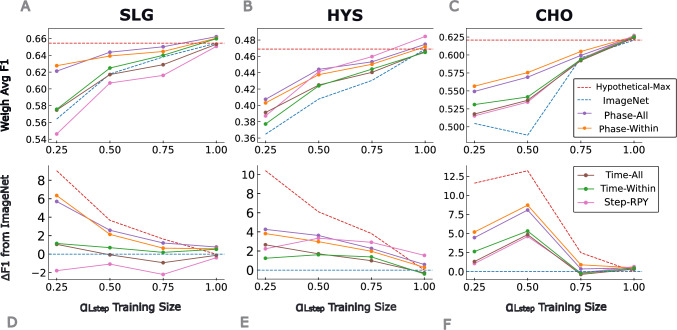
Effect of weakly supervised pre-training on surgical step recognition performance across three procedure types. **(A-C)** Weighted average F1-score for sleeve gastrectomy (SLG), hysterectomy (HYS), and cholecystectomy (CHO) across increasing fractions of annotated training data. The red dashed line represents the *Hypothetical Max* (full supervision, no weak pre-training), while the blue dashed line denotes the ImageNet-only baseline. Colored lines indicate different weak pre-training strategies. **(D-F)** Performance gain ($\varDelta F1$) from each pre-training strategy relative to the ImageNet-baseline, visualizing the effect of weak supervision under limited annotation

### Evaluation metrics

Model performance is measured using the **weighted average F1-score** across all step classes on the test set. We report:

- **Baseline:** ImageNet-pretrained model, directly fine-tuned on the target dataset.
- $\varDelta {\textbf {F1}}$: Improvement from weak pre-training relative to the baseline.
- **Hypothetical Max:** Performance when full annotations ($\alpha = 1.0$) are available and no weak pre-training is applied (baseline ImageNet-pretrained model).

Confidence intervals (95%) for all metrics are computed across the five random splits and paired two-sided t-tests are performed to determine statistical significance. Specifically, the pairs are formed by the random dataset splits which were held constant between the ImageNet-baseline and weak pre-training allowing for direct comparisons of performance. A Bonferroni correction is used when making multiple comparisons. Assuming *N* is the number of comparisons, a single asterisk (*) is used to indicate $0.01\le N\times p<0.05$, a double asterisk (**) indicates $0.001\le N\times p<0.01$, and a triple asterisk (***) indicates $N\times p<0.001$.

### Estimating label-efficiency in units of videos

To quantify label-efficiency, we report the *Number Needed to Label* (NNL): the number of additional labeled training videos the Baseline (ImageNet initialization, no weak pre-training) would require to match the performance achieved by a weakly pre-trained model at the same $\alpha$. For each target procedure *p*, let $F1^{\text {base}}_{p}(\alpha )$ be the Baseline weighted-F1 and $F1^{\text {pre}}_{p,L}(\alpha )$ be the weighted-F1 after weak pre-training using label type *L* (e.g., Phase-Within). We model the relationship between Baseline performance and label fraction with a simple linear fit over the four $\alpha$ points:

$$ F1^{\text {base}}_{p}(\alpha ) \;=\; a_{p} \;+\; b_{p}\,\alpha , $$

estimated by ordinary least squares. This is the linear fit to each ImageNet-baseline in Figure 2A, B, and C. We then evaluate the observed pre-training gain at a fixed low-label operating point $\alpha _0=0.25$:

$$ \varDelta F1_{p} \;=\; F1^{\text {pre}}_{p,L}(\alpha _0) \;-\; F1^{\text {base}}_{p}(\alpha _0). $$

The additional label fraction that the Baseline would need to achieve the same improvement, under the fitted slope $b_{p,s}$, is

$$ \varDelta \alpha _{p} \;=\; \frac{\varDelta F1_{p}}{b_{p}}. $$

Let $N^{\text {train}}_{p}$ denote the number of training videos for procedure *p* (i.e., $\approx 80\%$ of all videos for that procedure). The *Number Needed to Label* is then

$$ \textrm{NNL}_{p} \;=\; \varDelta \alpha _{p} \times N^{\text {train}}_{p}. $$

### Quantifying the effect of pre-training dataset Ssize

We conducted an additional experiment to isolate the effect of weak pre-training dataset size on model performance. For each target procedure (SLG, HYS, CHO), we fixed the fine-tuning data condition to a low-resource scenario ($\alpha = 0.25$) and varied the proportion of available phase-labeled data used for weak pre-training (0.25, 0.5, 0.75, 1.0) to understand its effect on the final Weighted Avg F1 score.

## Results

**Table 4:** Performance comparison on predicting surgical steps at $\alpha = 0.25$ across procedure types and weak label strategies

Pre-training Strategy	SLG	HYS	CHO
Phase-Within	6.35 [4.70–8.01]**	3.83 [2.25–5.40]**	5.19 [1.85–8.53]*
Phase-All	5.71 [4.70–6.73]***	4.27 [2.67–5.87]**	4.46 [2.31–6.60]**
Time-Within	1.16 [–0.29–2.61]	1.25 [–0.06–2.56]	2.63 [0.28–4.98]*
Time-All	1.07 [0.04–2.09]*	2.66 [–1.54–6.85]	1.31 [0.28–2.34]*
Step-RPY	–1.78 [–3.10––0.46]	2.24 [0.63–3.84]*	1.05 [0.18–1.93]*
Hypothetical Max	9.04 [7.24–10.85]	10.41 [8.71–12.12]	11.61 [0.78–22.43]

Each cell reports Mean $\varDelta$F1 [95% CI]. Asterisk indicates statistical significance relative to ImageNet-baseline. Bold numerical values indicate the best performance for that procedure type

We evaluated the impact of weakly supervised pre-training across three target procedure types–sleeve gastrectomy (SLG), hysterectomy (HYS), and cholecystectomy (CHO)–using varying levels of annotated step data ($\alpha \in \{0.25, 0.5, 0.75, 1.0\}$). Figure 2 illustrates the model performance for each pre-training strategy across these annotation conditions.

In the top row of Figure 2, we report the weighted average F1-score for surgical step recognition. The red dashed line represents the *Hypothetical Max*–the performance of a model trained with full supervision ($\alpha = 1.0$) but no weak pre-training. The second row displays the performance gain ($\varDelta \text {F1}$) over an ImageNet-pretrained baseline (blue dashed line at $y = 0$).

Table 4 reports the $\varDelta$F1 between the pre-training strategies and ImageNet-baseline at $\alpha = 0.25$. Though $\varDelta$F1 values are generally positive, note that specific pre-training strategies have differences in F1 values at $\alpha = 0.25$ that are statistically significant. The Phase-Within and Phase-All values are statistically significant across all procedure types in Table 4. Other pre-training strategies, like Step-RPY and Time-All, provide distinguishable benefit to specific procedure types while Time-Within only seems to provide measurable benefit to CHO. $\varDelta$F1 values at other $\alpha$ levels for pre-training strategies were *not* found to be statistically significant after correction for multiple comparisons. However, we note those configurations that continue to have positive values and exclude 0 from their 95% confidence intervals: i) Phase-Within for HYS and CHO at $\alpha = 0.5$, as well as, ii) Phase-All and Step-RPY for HYS at $\alpha = 0.5$.

### Phase-Based Pre-training

Models pre-trained with surgical phase labels–especially using data from the same procedure type (Phase-Within)–consistently outperformed the baseline in low-annotation settings ($\alpha = 0.25$). For instance, in SLG, Phase-Within led to a 6.35-point F1 improvement (95% CI: [4.70, 8.01]), closely approaching the *Hypothetical Max* (Table 4). Similar trends were observed for HYS and CHO, though with slightly lower gains. The composite Phase-All strategy also performed well having significantly different F1 values from baseline (Table 4 second row).

### Time-Based Pre-training

Pre-training with elapsed time labels yielded modest but consistent improvements across all procedures in low-annotation settings. For CHO, both Time-Within and Time-All improved F1-score significantly (2.6 and 1.3 points, respectively), while gains for SLG and HYS were more modest or non-significant.

### Cross-Procedural Step Pre-training

Pre-training on surgical steps from a different procedure type (Step-RPY) yielded mixed results. For HYS, this strategy provided a statistically significant gain (2.24 points, 95% CI: [0.63, 3.84]), outperforming phase-based models at higher annotation levels. For CHO, the improvement was modest (1.05 points), while for SLG, performance declined below the baseline.

### Estimated label-efficiency

We compute the *Number Needed to Label* (NNL) as defined in Section Estimating label-efficiency in units of videos and report this in Table 5. Note that the values for $\varDelta F1_{p}$ at $\alpha _0=0.25$ are reported in Table 4 along with confidence intervals. We use the values from the best weak label for each procedure type to compute $\varDelta \alpha _{p}$ and $NNL_p$ along with corresponding confidence intervals in Table 5. NNL expresses label-efficiency in operational units: an $\textrm{NNL}{=}20$ for procedure *p* at $\alpha _0{=}0.25$ means the Baseline would need approximately 20 *additional* labeled training videos from procedure *p* to match the performance achieved by weak pre-training at the same labeled fraction. We find that an additional 30–60 videos would need to be labeled to match the performance of the best weak-pretraining strategy at $\alpha _0=0.25$ across different procedure types. This value of the number of videos can be used to estimate the human labeling effort based on the time needed on average to annotate one video, as reported in other studies [27].

**Table 5:** Minimal NNL summary at $\alpha _0=0.25$ (best weak label per procedure). NNL: Number Needed to Label (videos) for the Baseline to match the weakly pre-trained model at the same $\alpha$

Procedure	Best weak label	$\varDelta$F1 [95% CI]	$N_{\text {train}}$	$\varDelta \alpha$ [95% CI]	**NNL** [95% CI]
SLG	Phase-Within	6.35 [4.70, 8.01]	68	0.53 [0.39, 0.67]	36 [27, 45]
HYS	Phase-All	4.27 [2.67, 5.87]	106	0.31 [0.19, 0.42]	33 [20, 45]
CHO	Phase-All	4.46 [2.31, 6.60]	263	0.25 [0.13, 0.37]	66 [34, 98]

**Notes.** $\varDelta$F1 is the absolute gain over Baseline at $\alpha _0{=}0.25$ (weighted-F1, points). $\varDelta \alpha$ is the label-fraction increase the Baseline would require to achieve the same $\varDelta$F1, computed from the split-wise slope of *F*1 vs. $\alpha$. $\textrm{NNL}=\varDelta \alpha \times N_{\text {train}}$. $L^\star$ is selected per procedure by highest mean $\varDelta$F1 (ties broken by smaller CI/greater robustness)

**Fig. 3:**
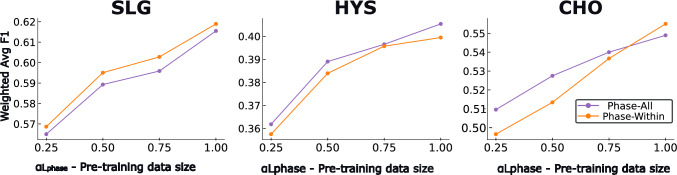
Impact of pre-training dataset size on downstream surgical step recognition performance. For each target procedure–SLG, HYS, and CHO–we fix the annotated step data to a low-resource condition ($\alpha$ = 0.25) and vary the amount of phase-labeled data (0.25, 0.5, 0.75, 1.0) used during weakly supervised pre-training. Results are shown for both within-procedure (Phase-Within) and cross-procedure (Phase-All) phase annotations

### Effect of Pre-training Dataset Size

To further understand the diminishing returns observed with weak supervision at higher annotation levels (Fig. 2), we isolated the effect of weak pre-training dataset size as described in Section Quantifying the effect of pre-training dataset Ssize. Results are shown in Figure 3.

Performance consistently improved with larger pre- training datasets for both Phase-Within and Phase-All strategies by a $\varDelta$F1 of 4-5 across procedure types, confirming that the size of the weak supervision source is a factor influence the performance on the target label. Interestingly, there is a moderate but apparent performance benefit of Phase-All for HYS and CHO, while SLG showed greater benefit from Phase-Within pre-training.

## Discussion

Taken together, our results indicate that weak supervision narrows the gap to full supervision under scarce labels: (i) phase-based pre-training yields consistent, cross-procedure gains; (ii) step- and time-based labels provide procedure-specific benefits; (iii) performance improves with larger pre-training sets; and (iv) the observed F1 gains translate into meaningful label-efficiency savings (NNL), i.e., fewer videos that require step annotation.

Weakly supervised pre-training substantially improves surgical step recognition when annotated data are limited. Phase labels—especially from the same target procedure or pooled across related procedures—produce the most reliable gains across all targets, supporting the use of weak labels to reduce manual annotation burden in surgical video pipelines and to accelerate model deployment for education and performance review.

Importantly, we achieved these improvements using a standard pre-training and fine-tuning paradigm on a relatively simple 2D CNN architecture. This suggests that further performance gains may be attainable through more advanced training regimes or model architectures, including temporal models or multi-modal inputs [12, 28].

The greatest benefit was observed with phase-based weak supervision, which yielded an average improvement of approximately 5 percentage points in F1-score (Table 4) across target procedures. This aligns with and extends the findings of Ramesh et al. [12], although their gains diminish in the absence of same-procedure phase labels. Our results highlight that even simple weak labels can unlock substantial improvements, comparable to several years of progress seen in benchmark datasets such as Cholec80 [26, 28].

Beyond average performance, weakly supervised models demonstrated improved robustness to cross-site or intra-procedural variability. For example, in HYS (Fig. 2E), performance of the baseline ImageNet model actually dropped as more annotated training data was added–likely due to increased variance from different surgical sites or styles [29]. In contrast, models pre-trained with weak labels remained stable or improved, suggesting that pre-training helps mitigate overfitting to noisy or heterogeneous training distributions. This robustness may help address known issues of model generalization across institutions [30].

The effectiveness of weak labels was not uniform. Phase annotations consistently generalized across procedures, likely due to their coarse-grained alignment with surgical workflow structure. In contrast, time-based labels provided moderate but procedure-specific benefit—most notably for CHO, a dataset with relatively linear procedures where elapsed time correlates with task progression. These results suggest that time-based labels may be more informative for datasets with more linear workflows. However, curated datasets can under-represent irregularities and real-world workflows may be less linear; thus, we frame time-label benefits as procedure- and dataset-dependent. Surprisingly, step pre-training from a different procedure (Step-RPY) significantly improved HYS performance, suggesting that some step-level visual or temporal patterns may transfer across seemingly unrelated procedures.

Arguably, this could reflect shared anatomical features, tools, or workflow components between radical prostatectomy and hysterectomy. We refrain from asserting causal mechanisms but note that pelvic procedures such as radical prostatectomy and hysterectomy share operating domain, instruments, and recurring workflow primitives (fine dissection, hemostasis, suturing). Such commonalities could provide transferable low-level features (e.g., energy-use signatures, instrument tip dynamics, camera motion patterns) that are subsequently specialized during fine-tuning on hysterectomy steps. By contrast, sleeve gastrectomy’s (SLG) upper-abdominal field and stapler-dominant steps likely reduce alignment with features learned from RPY, consistent with the weaker transfer we observe.

Table 5 summarizes the *Number Needed to Label* (NNL) at the low-label operating point $\alpha _0{=}0.25$. Across procedures, weak pre-training with phase information delivers sizeable annotation savings: for SLG, Phase-Within yields an additional label fraction of $\varDelta \alpha {=}0.53$ (95% CI [0.39, 0.67]). Under the same train-set size, the Baseline would require annotating 36 more SLG training videos (95% CI [27, 45]) to match that performance. HYS shows a similar pattern with Phase-All (NNL 33 [20, 45]; $\varDelta \alpha {=}0.31$ [0.19, 0.42]) and CHO with Phase-All (NNL 66 [34, 98]; $\varDelta \alpha {=}0.25$ [0.13, 0.37]).

Two observations follow. First, the *label-fraction* gains ($\varDelta \alpha \in [0.25,0.53]$) are comparable across procedures, indicating that phase-based weak pre-training consistently shifts the F1–$\alpha$ curve leftward at $\alpha _0{=}0.25$. Second, NNL scales with the size of the training pool $N_{\text {train}}$: CHO exhibits a larger NNL because the same $\varDelta \alpha$ multiplies a larger $N_{\text {train}}$ (263 vs. 68/106), making NNL especially useful for budgeting annotation effort at deployment sites with different data volumes. For example, the effort of labeling 66 additional cases (NNL for CHO in Table 5) can be roughly estimated to be $\sim$44 person-hours using the annotation time of $\sim$40 min per case as reported by Lecuyer et al for cholecystectomy videos [27].

These results suggest that while the change may only be “a few F1 points”, the observed $\varDelta$F1 translates into *dozens of additional videos* a baseline system would otherwise need to have labeled. We emphasize that NNL is an *interpretability* and *planning* tool rather than a replacement for standard effect sizes or significance tests; it depends on a local linear approximation of *F*1 vs. $\alpha$ and inherits uncertainty from both the fitted slope and the split-to-split variability (reflected in the reported CIs). Even with these caveats, NNL provides an actionable unit—videos—for comparing weak-label strategies and prioritizing annotation where it has the greatest marginal return.

As expected, the impact of weak supervision diminished with increased availability of annotated step data. This is likely due to the reduced ratio of pre-training data to task-specific training data. Our analysis (Fig 3) shows that maintaining a high ratio of pre-training data relative to fine-tuning data helps preserve the benefits of weak supervision. Interestingly, Step-RPY continued to yield gains even at full supervision ($\alpha = 1$), indicating potential for complementary learning beyond what is captured in the target dataset.

The weighted-F1 scores reported here are lower than state-of-the-art (SOTA) *precision/recall/accuracy* ranges reported on open benchmarks such as Cholec80, AutoLaparo, and MultiBypass140 (typically $\sim$65–95% depending on the publication and metric) [31–33]. This gap is expected given (i) task granularity (fine-grained *step* recognition vs. commonly reported *phase* recognition), (ii) metric differences (weighted-F1 vs. accuracy), (iii) data characteristics (our multi-site/private data and less curated distributions), and (iv) our explicit focus on low-label regimes (e.g., $\alpha \le 0.25$). Importantly, our goal was not to optimize absolute SOTA performance, but to conduct a *systematic* evaluation of *which* weak-label pre-training strategies improve step recognition across procedure types and *by how much*, emphasizing label-efficiency (NNL) under realistic annotation constraints. We view this contribution as complementary to SOTA model development; future work can combine the most effective weak-label strategies identified here with stronger temporal/multi-modal architectures to pursue both label efficiency and peak accuracy.

**Table 6:** Dataset provenance and availability (complements Table 1)

Proc. Type	Abbrv.	# Sites	Origin	Shareability
Sleeve Gastrectomy	SLG	1	Private	Aggregate statistics only
Hysterectomy	HYS	2	Private	Aggregate statistics only
Cholecystectomy	CHO	3	Private	Aggregate statistics only
Radical Prostatectomy	RPY	2	Private	Aggregate statistics only

This table documents site counts and shareability of the datasets used in this study. Aggregate statistics will be made available upon request

### Limitations and future work

One limitation of our approach is the computational overhead of evaluating multiple weak pre-training strategies per target task. Identifying the optimal dataset-label pair requires an exhaustive search, which may be prohibitive in practical deployments. Future work could explore automated selection mechanisms–e.g., using label similarity metrics, ontology alignment, or unsupervised correlation analyses–to guide efficient pre-training strategy selection. Additionally, joint training paradigms or meta-learning approaches may offer end-to-end optimization paths that reduce selection bias while retaining flexibility. Despite these costs, the modularity of our framework allows it to be applied to existing recognition pipelines without re-architecting the target model.

Moreover, our dataset includes videos from multiple clinical sites for *HYS*, *CHO*, and *RPY*, whereas *SLG* is single-site. We did not design experiments to isolate cross-site generalization (e.g., leave-one-site-out training/testing), so we refrain from drawing conclusions about robustness to inter-site distribution shifts. Nevertheless, the consistent gains from phase-based weak pre-training on procedures aggregated across sites suggest that coarser workflow supervision may help learn representations that are somewhat less sensitive to site-specific factors (e.g., optics, instrumentation, video encoding, and local protocols). Future work should (i) report per-site metrics, (ii) conduct stratified and leave-one-site-out evaluations with site as a grouping variable, and (iii) investigate multi-source/domain-generalization strategies (e.g., adversarial site-invariance, site-aware sampling, or multi-task objectives using site indicators) to quantify and strengthen cross-site transfer. Existing literature in this domain suggests that when deploying to a new site, collecting a small amount of site-specific step labels and combining them with phase-level weak pre-training offers a pragmatic path [34–37].

Additionally, since our study uses a 2D CNN at 1 fps without explicit temporal modeling, we plan to do future work to test whether the observed label-efficiency gains hold for temporal architectures along with temporal sampling rates higher than 1 fps. Here, we also use ImageNet initializations which are domain-general and may not be a strong foundation for analyzing surgical video. We also plan to test other initializations and architectures for video frame embeddings. Finally, there is a clear need to perform multi-source, multi-task pre-training across different procedure types and weak labels in order to investigate synergies in weak labels that may not contribute linearly to performance against the baseline. Specifically, there may be more data- or event-driven approaches to improving the representation of case progression (e.g. instrument installations, energy applications, or anatomy-in-view) instead of the monotonically increasing Time-Within and Time-All labels used here.

## Conclusion

This work demonstrates the potential of weakly supervised pre-training to improve surgical step recognition when annotated data is limited. By leveraging unannotated or heterogeneously labeled surgical videos–such as those labeled with phases, elapsed time, or cross-procedural steps–we show that substantial performance gains are achievable using simple models and standard training routines. Phase-based labels offer the most consistent improvements across procedures, while other weak labels exhibit procedure-specific value depending on workflow structure and anatomical similarity.

These findings suggest a scalable path forward for surgical video analysis in both education and skill assessment, particularly in data-constrained environments. By demonstrating the effectiveness of weak supervision across varied annotation regimes, this work lays the groundwork for integrating such methods into real-world surgical video review systems. Future work should explore joint modeling of weak labels, more advanced architectures, and automated strategies for selecting optimal pre-training signals based on task relevance and data availability. Additionally, incorporating online learning setups or user-in-the-loop annotation workflows may further reduce expert burden and enhance system adaptability over time.

## A Dataset details

See Table 6.

Details about how the annotation ontology was developed, how practicing surgeons were consulted, how it is annotated, and how the annotation framework is documented can be found in Mlambo et al. [9].
